# KIM-1 as a Biomarker in Genitourinary Neoplasms

**DOI:** 10.3390/cancers18081266

**Published:** 2026-04-16

**Authors:** Christos Veros, Aristotelis Bamias

**Affiliations:** 1 Medical School, National and Kapodistrian University of Athens, 11527 Athens, Greece; 2 Attikon University Hospital, 12462 Athens, Greece; abamias@med.uoa.gr

**Keywords:** renal cell carcinoma, kidney injury molecule-1, KIM-1, urine biomarkers, blood biomarkers, adjuvant immunotherapy, minimal residual disease, genitourinary cancers

## Abstract

Kidney Injury Molecule-1 (KIM-1), a type I transmembrane glycoprotein, is emerging as a promising biomarker in the landscape of genitourinary malignancies. Initially it was described for its role in renal tubular injury, but it is currently being studied for its expression in various neoplasms, particularly renal cell carcinoma, where it correlates with tumor grade, stage and prognosis. Recent studies have demonstrated its potential usefulness as a non-invasive diagnostic tool through urinary and plasma/serum studies, offering a valuable adjunct to imaging and pathology studies. KIM-1 may also play a role in urothelial cancer, although its specificity and relevance in this context are not clearly established yet. The following review presents our current knowledge on the biology of KIM-1, its expression patterns across various genitourinary tumors and its clinical implications in early detection, prognosis and treatment monitoring. We also explore the limitations and future directions regarding the integration of KIM-1 into precision oncology approaches.

## Simple Summary

Urological cancers include renal cancer, bladder cancer, prostate cancer, penile cancer and testicular germ cell tumors. The majority of patients occasionally face significant uncertainty during the course of their disease, regarding recurrence risk and treatment benefit.. Kidney injury molecule-1 (KIM-1) is a protein that can be possibly used as a biomarker to stratify patients in prognostic categories and offer valuable information about treatment response. It can be measured in urine, blood and tumor specimens. While it was first studied as a biomarker indicative of kidney injury, it is also overexpressed in renal cancer and more rarely in other tumors of the genitourinary system. This review focuses on KIM-1 function, detection and potential clinical utility, reflecting its usefulness in detecting urological cancers, estimating recurrence risk after surgery and potentially guiding adjuvant immunotherapy. We also discuss limitations such as reduced cancer specificity in patients with chronic kidney disease, differences between laboratory assays and the need for prospective validation before routine clinical use.

## 1. Introduction

Genitourinary cancers are biologically diverse and clinically heterogeneous malignancies. They include renal cell carcinoma (RCC), urothelial carcinoma of the bladder and upper urinary tract, prostate adenocarcinoma, penile cancer, testicular cancer and other rarer entities. Among the aforementioned neoplasms, RCC is notable both for its incidence and for the challenges it conveys regarding diagnosis and postoperative surveillance. Cross-sectional imaging has increased the detection of renal incidentalomas. However, while some surgically removed renal masses are benign, many small RCCs are known to behave indolently [1]. On the other hand, a subset of patients with presumably localized RCC relapse after nephrectomy, highlighting the yet unmet need for new biomarkers that improve our ability to help individualize the recurrence risk beyond clinical and pathologic parameters and finally support earlier detection of recurrence or play a role in the selection of adjuvant therapy [2].

Current treatment strategies for renal and urothelial cancers have considerably evolved in the last decade, and they rely on multimodal approaches that reflect the biological diversity and clinical behavior of these tumors. The mainstay for the management of localized RCC remains surgical resection, but adjuvant therapy with the immune checkpoint inhibitor (ICI), pembrolizumab, is now the standard for high-risk localized tumors. For advanced RCC, treatment has shifted towards the combination of targeted therapies, namely anti-VEGF tyrosine kinase inhibitors (TKIs) such as axitinib, lenvatinib and cabozantinib, together with immune checkpoint inhibitors (ICIs), pembrolizumab and nivolumab. For first-line approaches, dual immunotherapy with anti-PD1 and anti-CTLA-4 agents (combination of nivolumab and ipilimumab) or ICI-TKI in combination represents the current standard. Radiotherapy plays a limited role in RCC, mostly for palliative reasons or for the stereotactic treatment of oligometastatic disease [3].

Regarding urothelial carcinoma (UC), non-muscle invasive, high-risk tumors are treated with intravesical therapy (e.g., BCG). In muscle-invasive disease, perioperative platinum-based chemotherapy and ICIs have recently become the standard of care, preceding and following surgery. In cases where a bladder-sparing approach is preferred, optimal transurethral resection of bladder tumor (TURBT) followed by concurrent chemoradiotherapy can produce outcomes similar to perioperative therapy and radical cystectomy in selected patients. Finally, the introduction of ICIs and antibody–drug conjugates has revolutionized prognosis in metastatic UC. In addition, FGFR inhibitors, such as erdafitinib, are now used for molecularly selected patients, thus introducing for the first time targeted therapy in our armamentarium against urothelial cancer. These evolving therapeutic landscapes highlight the increasing importance of integrating reliable biomarkers, such as KIM-1 and ctDNA, into diagnostic, prognostic, and treatment frameworks [4,5,6].

Kidney injury molecule-1 (KIM-1), also termed Hepatitis A virus cellular receptor A (HAVCR1) or T-cell immunoglobulin and mucin domain 1 (TIM-1), is a type I transmembrane glycoprotein initially characterized as a laboratory marker of injury of the epithelium of renal proximal tubules [7]. Its biology is gaining attention in genitourinary oncology because it is not only produced in proximal tubules after injury; it is also frequently expressed in RCC arising from tubular epithelium, especially clear cell and papillary renal cancer, while its extracellular domain undergoes proteolytic shedding, enabling its easy detection in urine and blood [7,8,9]. A growing body of clinical evidence supports an association between blood or urinary KIM-1 and incidence of RCC, tumor burden and its prognosis, including recurrence risk after nephrectomy and its potential usefulness as a biomarker in the adjuvant immunotherapy setting. A common problem clinicians face when treating patients with urothelial cancer is the fact that current imaging studies have limited sensitivity regarding the detection of microscopic or early recurrent disease, a pitfall that often leads to delayed identification of relapse. Pathologic staging may also be imperfect, since tumor heterogeneity can result in underestimation of aggressive biological behavior. These limitations contribute to a clinical paradox of some patients being overtreated for indolent tumors, while others with high-risk disease receive insufficient surveillance or delayed therapy [2,10,11,12]. Recent reviews further emphasize the potential role of KIM-1 as a biomarker in RCC as well as in other genitourinary neoplasms. This review depicts the molecular and cellular biology of KIM-1, its patterns of expression across different genitourinary tumors, the available evidence regarding its measurement in urine and blood in patients, its possible clinical implications and considerations towards its classification as an emerging biomarker for clinical use.

## 2. Materials and Methods

The following narrative review aims to present existing knowledge on the clinical significance of KIM-1 in renal cell and other genitourinary cancers, with a specific focus on its molecular structure, its expression across different biological entities and its emerging role as a biomarker in diagnosis, prognosis and treatment monitoring. A structured search of existing literature was pursued using four electronic databases—PubMed, Scopus, Google Scholar, ResearchGate and ScienceDirect—between October 2025 and January 2026, using a double-blinded process. We selected full publications on KIM-1; the search strategy included a combination of medical terms, such as “Kidney Injury Molecule-1”, “KIM-1”, “renal cell carcinoma”, “RCC”, “urological cancers”, “bladder cancer”, “urothelial cancer”, “germ cell tumors”, “prostate cancer”, “biomarker”, “diagnosis”, “prognosis”, “immunotherapy”, “treatment monitoring”, “urinary biomarkers”, “blood biomarkers” and “liquid biopsy” among others. Eligible scientific papers were chosen after meeting the following conditions: (i) publication in peer-reviewed journals written in the English language, (ii) inclusion of humans diagnosed with genitourinary neoplasms and particularly renal cell cancer and (iii) employment of scientifically robust designs, such as prospective or retrospective studies, randomized controlled trials and systematic reviews. The type of biological sample used in these studies to measure KIM-1 should be blood or urine. Studies were excluded in case they met the following criteria: (i) in vitro, preclinical, or animal-based studies, (ii) lack of focus on KIM-1 within the context of urogenital cancers, (iii) non-peer-reviewed articles, including conference abstracts, opinion pieces or editorials, (iv) publication in gray literature or (v) absence of full-text availability. This research approach was employed to ensure a high level of scientific credibility and to enhance a comprehensive and meaningful synthesis of the existing scientific material on KIM-1 as a biomarker in urogenital cancers.

## 3. Results

### 3.1. Molecular Biology of KIM-1 and Its Detection in Biological Fluids

**Figure 1:**
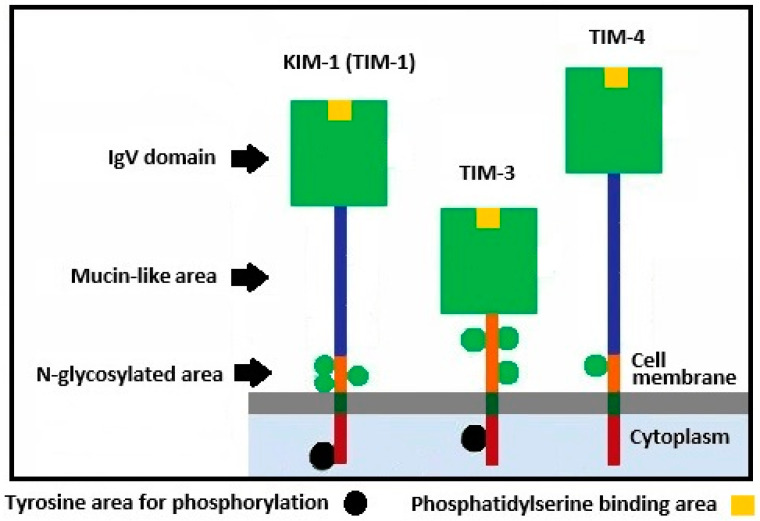
KIM family members.

KIM-1 (Gene ID: 26762) is located on the long arm of chromosome 5 at locus 5q33.3 and contains 11 exons. Its variants can be generated as a result of alternative mRNA splicing and can contain from 334 to 401 amino acids [13,14,15]. The molecular mass of fully glycosylated KIM-1 reaches 104 kDa [14]. KIM-1 is localized on the cellular plasma membrane and has three domains: an extracellular, a transmembrane and a cytoplasmic one (Figure 1) [13,16]. The extracellular domain of KIM-1 consists of a globular structure similar to the variable fragment of immunoglobulins (IgV), a mucin-like sequence and a short peptide area. A key feature of the IgV-domain structure of KIM-1 is the existence of a hydrophobic “pocket” (a binding site which depends on metal ions), which functions as a binding site for a signaling phospholipid, phosphatidylserine (PS) [13,17]. Normally, PS is found on the internal side of the cellular plasma membrane, and when apoptosis is induced, it moves to the external side of the plasma membrane. It serves as a signal molecule for epithelial cells and macrophages and leads to the absorption of dead cells [13,18]. Because of its ability to interact with PS, KIM-1 in the epithelial cells can mediate the in situ removal of cellular debris when tissue damage is present [3,19]. Its glycosylated mucin-like domain, which forms the biggest part of KIM-1, includes tandem repeats of amino acids and several sites of O-glycosylation. This domain has no specific functional properties; however, we can assume that the organization of this area is important for the interaction of KIM-1 with its ligands [13,16,20]. As a result of proteolytic cleavage, the extracellular portion of KIM-1 is shed from the cell and a free form of KIM-1 circulates and can be identified in urine and blood serum [13,14,21]. As such, KIM-1 finally owns a cytoplasmic domain which is a variable short polypeptide, a fact which raises the possibility that KIM-1 is also involved in intracellular signaling [13,14,20]. Figure 1 below depicts the members of the KIM family.

### 3.2. Biology of Kidney Injury

Acute kidney injury (AKI) is usually the result of ischemic shock or acute cardiovascular insufficiency, septic or toxic injury of the kidneys and urinary tract obstruction [13]. The most common cause of AKI is ischemia [13,22] which leads to death and shedding of tubular epithelial cells from the basal lamina. AKI is sometimes reversible due to the fact that the epithelium of kidney tubules possesses high regeneration ability. After shock, viable cells undergo epithelial–mesenchymal transition, proliferate and move to the affected areas where they reassume their expected differentiated epithelial phenotype [13,23].

In renal injury, KIM-1 is strongly produced by proximal tubular epithelial cells and correlates with the whole process of damage and repair. When the epithelial cells of the kidney die, a soluble form of KIM-1 together with fluid enters the interstitium and as a result the bloodstream [24]. Higher concentration of KIM-1 in blood and in urine reflects kidney tubular injury [25]. Persistent production of KIM-1 in chronic injury has been associated with maladaptive pathways (including fibrosis), which is an important factor to take into consideration when interpreting urine KIM-1 in cases with chronic kidney disease (CKD).

As a result, KIM-1 is an ideal biomarker of proximal tubule epithelial injury [26]: in the normal kidney, KIM-1 is expressed in traces. In kidney injury of any etiology, the synthetic activation of KIM-1 in the damaged cells and its increase in expression are a well-established phenomenon. Shedding of KIM-1 from the damaged cells results in its considerable increase in urine and/or blood. The increased concentration of KIM-1 in urine (uKIM-1) is a more sensitive indicator of AKI compared to the reduction in creatinine clearance or albuminuria.

In cancer, the mechanism of KIM-1 production and shedding is similar and is a result of the destruction of normal tissues, because of phenomena like ischemia or mechanical damage of normal kidney tissue from the expanding neoplasm. KIM-1 has also been found to play an important role in cell adhesion, epithelial remodeling, inflammatory signaling and microenvironmental interactions, providing a plausible biological basis for the association between high KIM-1 expression and aggressive RCC phenotypes in a subset of patients [11,13].

### 3.3. KIM-1 and Liquid Biopsy

KIM-1 shedding makes it appealing as a biomarker. If KIM-1 is highly expressed in tumor cells (or tumor-adjacent tissues), shedding can elevate its soluble form in blood and urine, allowing repeated, minimally invasive monitoring. However, because non-malignant types of injury also increase KIM-1 levels, a clinically useful biomarker strategy must incorporate clinical context (renal function, inflammation, and obstruction) and analytical validation [21]. The following table (Table 1) presents various advantages and limitations of KIM-1 sampling types.

### 3.4. KIM-1 Expression Across Genitourinary Malignancies

Evidence for KIM-1 expression is strongest for renal cell cancer. Its expression is highest in clear cell RCC and papillary RCC, with substantially lower expression in chromophobe RCC and benign oncocytoma. Furthermore, KIM-1 is expressed in urothelial, prostate and germ cell tumors, but its role in these cancers is less established to date, since its expression is less consistent. This histology-driven pattern is clinically important because it possibly predicts where biofluid detection is most likely to be helpful in terms of its function as a biomarker [1,7,12,21].

#### 3.4.1. Clear Cell Renal Cancer

Early-stage renal cell cancer presents a critical challenge for intervention, since identifying reliable biomarkers for this stage can significantly impact decisions regarding management. KIM-1 theoretically plays an important functional role in the pathogenetic mechanisms of renal cancer and disease progression. According to many in vitro studies, the expression of KIM-1 in the human umbilical vein endothelial cells (HUVEC) transfected by *HAVcr-1* leads to an increase in their disintegration and also increases their sensitivity to the action of hepatocyte growth factor, resulting in the destruction of intercellular contacts [7,13]. KIM-1 interacts with Rho GTPase, a molecule which regulates the assembly of the cytoplasmic ZO-protein complex, enhancing the structure of tight cellular junctions. This mechanism can lead to the disintegration of tumor cells and enable metastasizing potential. TIM-1-mediated regulation of nuclear receptor Nur77 degradation leads to the inhibition of proapoptotic signals in the cells of renal carcinoma. This mechanism maintains the integrity of the tubular epithelium of the kidney in ischemic states and can contribute to the increased survival of the tumor cells. According to Cuadros et al., overexpression of KIM-1 in malignant cells in vitro correlates with greater proliferation and increased IL-6 production. IL-6 expression directly correlates with the volume of KIM-1 shedding from the tumor cells. It is believed that the extracellular domain of KIM-1 can activate the KIM-1/IL-6/STAT-3 signaling axis via an autocrine or paracrine mechanism. Irrespective of KIM-1 expression in the tumor cells, an increase in its synthesis is also observed in the epithelium of normal kidney cells, which surround the malignant tumor. This might be a consequence of tissue damage because of compression and ischemia, which are caused by the progressive growth of the tumor. The increase in KIM-1 expression in chronic hypoxia sustains low-grade inflammation and the extracellular KIM-1 area can interact with the components of tumor stroma, such as endothelial cells, lymphocytes and tumor-associated myeloid cells. The observed inflammation acts as a source of cytokines and growth factors and stimulates the proliferation of cancer cells. Furthermore, it leads to neoangiogenesis and attracts immune suppressor cells, a phenomenon which leads to the suppression of the immune system, inhibiting lymphocytes. As a result, abnormal KIM-1 expression in RCC and the surrounding normal tissue can contribute to the formation of a cancer-tolerant microenvironment, enabling cancer cells to escape from immune surveillance [13].

Several studies have proven that elevated plasma or urine levels of KIM-1 are a sign of the existence of renal cell cancer even before being clinically diagnosed. Scelo et al. measured KIM-1 levels in individuals before being diagnosed, which allowed for earlier detection of RCC [21,27]. The incidence rate ratio (IRR) of RCC was 1.71 for every doubling of plasma KIM-1 concentration. This demonstrates a proportionate connection between KIM-1 levels and increased risk. Karmakova and Han et al. showed that KIM-1 expression is much higher in clear cell renal cancer than in other renal malignancies. In chromophobe RCC and benign renal oncocytomas, the expression of KIM-1 is rare [13,21,28]. The differential expression of KIM-1 underscores its specificity as a biomarker regarding clear cell renal cancer. KIM-1 can be measured in urine and blood. Elevated levels of this biomarker in these bodily fluids have been linked to RCC, especially in the post-diagnostic setting. KIM-1 is a non-invasive biomarker. It can be utilized for early-stage RCC and can be evaluated using urine or blood samples for testing. This fact allows for early diagnosis and serial monitoring of the malignancy [21,29,30].

The sensitivity and specificity of KIM-1 as a biomarker have been widely investigated. It has been shown that although KIM-1 is considerably specific, its sensitivity presents fluctuations. In urine samples from RCC patients, the sensitivity of KIM-1 in some cases surpasses 90%. However, this can be affected by different variables, including the size of the sample and the methodology used for detection.

Beyond diagnosis and disease monitoring, KIM-1 also has prognostic value. There is a direct correlation between tumor grade or size and KIM-1 levels. More aggressive tumors express higher levels of KIM-1. This proves its role in predicting the trajectory of the disease and allows for the stratification of patients based on risk evaluation [29,30]. Elevated KIM-1 levels have been linked with poor survival. Thus, measuring KIM-1 levels in urine offers a non-invasive method to prognostically stratify renal cancer and this could represent a valuable clinical instrument [31,32].

One major advantage of using KIM-1 as a prognostic biomarker is the fact that it reflects the biological behavior of the tumor. Zhang et al. showed that the levels of KIM-1 in the urine are affected by many factors, such as the level of production of the biomarker by tumor cells, tumor size and the way through which it is excreted into the urine [10,21]. This renders KIM-1 a dynamic biomarker that enables real-time assessment of the status and progression of the tumor. Integrating KIM-1 into clinical practice offers a tool for the monitoring of disease progression. Urine sampling is noninvasive, thus allowing for repeated measurements, providing a longitudinal assessment of the patient’s condition. This is useful especially in detecting early signs of subclinical recurrence or progression, enabling timely interventions before progression is observed in imaging studies [32,33].

KIM-1 may also be employed in choosing the most appropriate therapy for the patient. The rationale behind this is the fact that biomarker-based adjuvant strategies have the goal to treat patients who are most likely to experience disease progression, while simultaneously avoiding overtreatment of lower-risk patients. Based on the IMmotion 010 trial, patients with high baseline levels of plasma KIM-1 had an increased risk of disease recurrence regardless of treatment. This subset of patients showed increased clinical benefit after receiving adjuvant atezolizumab over placebo, despite no proven clinical benefit in the intention-to-treat (ITT) population. Dynamic changes in KIM-1 during treatment were linked to recurrence risk, which allows us to hypothesize that KIM-1 in the plasma can function as a biomarker for minimal residual disease evaluation in RCC. These observations are also consistent with previous data from the metastatic setting in other studies which show that a decrease in serum levels of KIM-1 after nivolumab (CheckMate 009) or nivolumab plus ipilimumab (CheckMate 214) is associated with better patient outcomes, including response in imaging studies, progression-free survival and overall survival [34,35,36].

Table 2 presents some representative clinical studies on KIM-1 in renal cell cancer (Table 2).

#### 3.4.2. Urothelial Cancer

Data regarding the diagnostic usefulness of urinary KIM-1 in urothelial carcinoma remain relatively sparse and their interpretation is complicated. Since KIM-1 is a highly sensitive marker of proximal tubular injury, its elevations in urine samples can arise from a wide spectrum of non-malignant etiologies, including obstruction, hydronephrosis, ischemia and inflammatory injury. These secondary effects can generate a misleading biomarker profile that mimics a tumor-associated picture, thereby reducing specificity and creating what can effectively be described as a “false cancer signal”, creating a confusing picture. This overlap is especially relevant in patients with ureteral obstruction or chronic irritation, where tubular stress may be substantial even in the absence of malignant transformation [21].

Attempts to use urine KIM-1 to differentiate between urothelial carcinoma and renal cell carcinoma have produced mixed results. Several exploratory studies have shown that while KIM-1 levels tend to be higher in RCC due to direct tumor expression and shedding, a subset of urothelial carcinoma cases (particularly patients with concomitant obstruction or renal pelvis involvement) also exhibit elevated concentrations. As a result, when KIM-1 is incorporated into multimarker panels or is combined with clinical variables, such as imaging findings or cytological studies, urine KIM-1 may provide some diagnostic value. Current evidence therefore supports its role as a potential adjunctive tool rather than a single test, contributing additional context, but lacking the specificity required for definitive differentiation between urothelial and renal cancers [7].

#### 3.4.3. Prostate Cancer

Evidence for KIM-1 as a prostate cancer biomarker remains exploratory. Its specificity and clinical utility are not established compared with already established prostate biomarkers like PSA and imaging pathways. KIM-1 is not routinely elevated in prostate adenocarcinoma, and it is not used clinically for diagnosis, prognosis, or monitoring. KIM-1 expression in prostate epithelial tumor cells themselves is generally low or absent [39].

#### 3.4.4. Testicular Germ Cell Tumors

A study conducted by Sangoi et al. on 100 germ cell tumor specimens immunohistochemically measured the expression of KIM-1 in 10 of 21 (48%) embryonal carcinomas and 4 of 8 (50%) yolk sac tumors. Expression was variable in both tumor types. There are no clinical studies evaluating KIM-1 as a biomarker in germ cell tumors and, as a result, this specific biomarker has zero clinical utility in this context [40]. Encountering KIM-1 in patients with germ cell tumors might probably signify cisplatin-induced renal injury or hydronephrosis and tubular injury from retroperitoneal lymphadenopathy.

### 3.5. Limitations of KIM-1 as a Biomarker

KIM-1 has demonstrated its potential as a biomarker for the diagnosis and prognosis, mainly of renal cancer and to a lesser extent in urothelial cancer. However, there are several limitations warranting consideration. Firstly, a notable limitation is the observed variability of the concentration of KIM-1 across different individuals, a fact which complicates the establishment of a universally accepted threshold for clinical application. The concentration of KIM-1 in serum, plasma or urine can vary significantly. This variability requires precise validation of KIM-1 concentration levels across different populations. This approach will guarantee increased accuracy in risk assessment. A second limitation is the probable impact of co-existent kidney injury on KIM-1 measurement [13,21]. This can result in false positive results, since elevated KIM-1 concentrations could be falsely interpreted as indicative of renal cancer when they should be attributed to other renal diseases. The timing of the KIM-1 measurement is also a challenge. KIM-1 levels can vary based on the timing of measurement in relation to disease progression and treatment choices [21,33]. For example, it has been consistently observed that KIM-1 levels drop significantly after nephrectomy, which means that the timing of sample collection is crucial for correct and accurate interpretation [21,27]. There are also genetic differences among individuals, which can impact KIM-1 expression. Also, other comorbid conditions such as diabetes mellitus, chronic kidney disease and hypertension, may create confusion regarding the interpretation of KIM-1 levels in non-oncologic patients. Last but not least, inconsistencies across different studies stem from differences in proteolytic shedding, sample storage and handling and the glycosylation level of the protein [14,21,27]. Addressing these limitations is essential if we want to ensure the reliability of the clinical application of KIM-1 across urological cancers.

## 4. Future Directions

Future progress in establishing KIM-1 as a clinically applicable biomarker will depend on several factors. Firstly, large, multicenter cohorts with standardized preoperative plasma, serum, or urine specimens are essential to validate the usefulness of circulating KIM-1 to discern between malignant and benign renal or urothelial lesions. These studies should also incorporate imaging studies and biopsy data to quantify whether KIM-1–guided decision-making can be used as a clinical tool to distinguish the benign or malignant nature of renal and urothelial masses. Further analyses will clarify if integrating KIM-1 into risk stratification is a cost-effective approach.

Moreover, the postoperative setting offers an important opportunity to evaluate KIM-1 as a marker of minimal residual disease (MRD). Prespecified cohorts should compare postoperative KIM-1 levels and dynamics with already used prognostic models. These studies will possibly determine whether KIM-1 is useful in predicting the risk of disease relapse and whether dynamic changes in its concentration can be utilized as early signals of recurrence before radiographic progression. In this case, integration with modern liquid biopsy platforms—including other molecules such as circulating tumor DNA (ctDNA), circulating proteins and immune signatures—could enable more accurate MRD assessment, improving sensitivity and specificity beyond KIM-1 alone. Such models can overcome the limitations of single biomarkers and improve diagnostic accuracy, risk stratification, and early relapse detection.

Furthermore, prognostic association should also be translated into clinical usefulness. This will require KIM-1-guided treatment trials, which will test whether KIM-1 can identify patients most likely to benefit from systemic therapy. In these trials, KIM-1 can influence the choice of treatment regimen, thereby directly testing biomarker-guided therapeutic strategies.

Finally, for KIM-1 to be clinically applied, standardized measurement methods should be adopted. This is essential for level evaluation, comparability among different laboratories and eventual integration of KIM-1 testing into clinical practice. Incorporating these specific study designs and standardization procedures will help transform KIM-1 from a promising biomarker into a validated tool in precision oncology.

## 5. Conclusions

KIM-1 represents one of the most attractive biomarker candidates in contemporary genitourinary oncology, particularly for renal cell and urothelial carcinoma. Its consistent overexpression provides the foundation for its reliable detection in both plasma/serum and urine. This is especially valuable in a malignancy where non-invasive biomarkers have been lacking so far. Current evidence suggests that circulating KIM-1 may improve preoperative renal and urothelial tumor risk stratification by helping distinguish malignant from benign lesions, enhance postoperative estimate of recurrence risk and support surveillance by detecting dynamic changes in tumor burden. Early exploratory analyses in the adjuvant immunotherapy setting further show a promising role for KIM-1 in identifying patients with residual disease who may derive benefit from systemic therapy.

Despite these encouraging data, several limitations need to be overcome before KIM-1 can be integrated into routine clinical practice. Prospective, multicenter validation studies are needed to confirm its diagnostic and prognostic usefulness across different populations and clinical contexts. Also, we should establish standardized platforms and validated thresholds to ensure consistency across laboratories. Because KIM-1 is a sensitive marker of tubular injury in general, cautious control of other parameters, such as acute or chronic kidney dysfunction, is essential to avoid misinterpretation in both research and clinical settings. Only through such efforts will we be able to determine whether KIM-1 can move from a promising research biomarker to a clinically relevant tool that improves decision-making in genitourinary cancers.

## Figures and Tables

**Table 1:** Practical comparison of various types of sampling for KIM-1 measurement.

Specimen	Advantages	Key Limitations	Best-Fit Clinical Question
Urine (uKIM-1) with or without creatinine normalization	Non-invasive, frequent sampling; can fall after nephrectomy	Strongly confounded by tubular injury, CKD, infection, and obstruction; dilution effects; needs normalization.	Serial monitoring in selected patients; adjunct evaluation; research
Plasma/Serum (pKIM-1/sKIM-1)	Standardized collection; promising preoperative discrimination and postoperative risk stratification	Assay-platform variability; need validated cutoffs; systemic/renal confounding	Preoperative risk stratification; postoperative MRD-risk; adjuvant-selection research
Combined urine + blood	Complementary biology; may improve robustness	More complex; requires integrated models	High-risk pathways; prospective studies; clinical trials

**Table 2:** Selected KIM-1 clinical studies relevant to RCC.

Study	Design/Population	Specimen	Primary Signal	Translational Takeaway
Xu et al. Post hoc analysis of Checkmate-214 [36]	Advanced renal cell carcinoma	Serum	Extent of reduction in KIM-1 is associated with long-term efficacy	Preoperative risk stratification tool
ECOG-ACRIN E2805 analysis [2]	Post-nephrectomy localized RCC	Plasma	Higher pKIM-1 associated with worse outcomes	Postoperative risk refinement
Ann. Oncol. 2025 biomarker analysis [34]	Adjuvant immunotherapy context	Blood	Prognostic association, subgroup signals	Hypothesis for biomarker-guided adjuvant selection
Mijugković et al. 2016 [30]	Clear RCC before/after surgery	Urine	High in pre-operative setting, post-op decrease	Urine monitoring potential
Sergeeva et al. 2021 [37]	RCC vs. controls, normalization analysis	Urine	uKIM-1/Cr improves interpretability	Normalization matters
He et al. 2025 [38]	Systematic review/meta-analysis	Liquid-based	Diagnostic/prognostic value across studies	Supports KIM-1 value as biomarker, highlights heterogeneity

## Data Availability

No new data were created or analyzed in this study.
